# Ghrelin Levels in Children with Constitutional Delay of Growth and Puberty

**DOI:** 10.4274/jcrpe.v2i3.117

**Published:** 2010-08-05

**Authors:** Tolta Altuğ Şen, Damla Gökşen Şimşek, Şükran Darcan, Mahmut Çoker

**Affiliations:** 1 Afyon Kocatepe University, Faculty of Medicine, Department of Pediatrics, Afyonkarahisar, Turkey; 2 Ege University Faculty of Medicine, Department of Pediatrics and Pediatric Endocrinology, İzmir, Turkey; +90 272 214 20 65/3014+90 272 246 33 22tolgasen69@yahoo.comAfyon Kocatepe University Faculty of Medicine, Department of Pediatrics / Pediatric Endocrinology, 03200, Afyonkarahisar, Turkey

**Keywords:** ghrelin, IGF−1, constitutional delay of growth and puberty

## Abstract

**Objective**: In this study, we aimed to show the role of ghrelin in growth delay in children with constitutional delay of growth and puberty (CDGP).

**Methods**: Thirty male children with CDGP constituted the study group and fifteen healthy children with normal growth of similar ages−the control group. In both groups, fasting and postprandial plasma ghrelin levels, serum insulin−like growth factor−1 (IGF−1) and IGF−binding protein−3 (IGFBP−3) levels were determined.

**Results**: There were no differences in fasting and postprandial ghrelin levels (824.23±523.46 pg/mL and 447.26±259.92 pg/mL, respectively) in children with CDGP compared to the levels in the control group (687.38±481.43 pg/mL and 365.59±260.43 pg/mL, respectively; p>0.05). Differences in fasting and postprandial ghrelin levels were also similar in the two groups (394.44±369.10 pg/mL and 346.55±338.67 pg/mL, respectively; p>0.05). Serum IGF−1 levels were significantly depressed in children with CDGP compared to those in the control group (239.5±83.95 ng/mL and 339.20±63.08 ng/mL, respectively; p<0.05).

**Conclusion**: Decreased appetite and feeding problems in children with CDGP were not related to depressed ghrelin levels. In addition, ghrelin levels did not increase to compensate for the decreased appetite and feeding problems in CDGP.

**Conflict of interest:**None declared.

## INTRODUCTION

Ghrelin is an important gastrointestinal hormone, which maintains the energy homeostasis by delivering neuroendocrine signals from the stomach to the brain (1, 2). Suboptimal nutrition, genetics, aberrations in insulin−like growth factor−1 (IGF−1) and growth hormone (GH) axis have all been accused as responsible factors in the etiology of constitutional delay of growth and puberty (CDGP) (3, 4, 5, 6). We hypothesized that defects in both GH secretagogue and orexigenic properties of ghrelin may contribute to delayed growth. Studies on the possible role of ghrelin on growth are limited in number. In this study, we compared plasma ghrelin levels of normally growing children with those of children with CDGP.

## MATERIALS AND METHODS

**Study Group**

The study was conducted on thirty male children with CDGP between the ages of 10.5 and 15.5 years. All children were born at term with birth weights appropriate for gestational age and had not suffered from any significant health problems. Fifteen healthy male children of similar ages constituted the control group. In 19 out of the 30 (63.3%) boys, the family history was positive for CDGP, and none of subjects had a history of chronic illness, important surgical intervention or trauma. Informed consent was taken from the children and their parents and the study was approved by the local ethics committee.

Weight and height measurements were done by the same medical staff early in the morning. For the weight measurement, a device (SECA^©^) with an accuracy of 0.1 kg and maximum capacity of 150 kg was used. For the height measurement, Harpenden stadiometer^©^ (Holtain Instruments Ltd, U.K) with an accuracy of 0.1cm was used, in which, child was in erect position with heels, buttocks and scapulae in contact with the device. Body mass index (BMI) was calculated using the standard formula [BMI= Weight (kg) / Height^2^ (meter)]. Evaluations of weight, height and BMI, and their SDS calculations were performed according to the charts prepared by Neyzi et al (7) for Turkish children. Pubertal staging was determined by Tanner’s classification (8). Prader’s orchidometer was used for measurement of testicular volume (9).

The heights of the children with CDGP were below the third percentile (−2 SD); upper/lower segment ratios were appropriate according to the standards (10). Bone age was assessed according to the Greulich−Pyle atlas (11). The bone ages of the children with CDGP were delayed with respect to their chronological ages by at least two years. Their physical examination findings were normal. The parents of the children with CDGP had normal height SDS values.

The children with CDGP were followed for at least one year in our clinic. Their target heights were similar to their predicted adult heights.

Daily energy and protein intake for the children in both groups were calculated by a dietician using a 3−day diet record. No children had overt malnutrition. Complete blood count, erythrocyte sedimentation rate, routine chemical analysis including renal and liver function tests, urine analysis and thyroid function tests were all normal for both groups. Antigliadin and antiendomysium antibodies were negative in all children, excluding celiac disease.

Blood samples for ghrelin levels were obtained from antecubital veins at 08.00 a.m. after a twelve−hour fast. Then, they received a standard breakfast arranged according to their sex, age, height and weight. Two hours after consuming that breakfast, blood samples were taken for the postprandial ghrelin levels. Ethylenediamine tetraacetic acid (EDTA) was used for anticoagulation of the blood samples. After centrifugation, HCl (in 1/10 ratio) and 10−15 μL aprotinin (protease inhibitor) were added to the samples for preservation. All samples were kept at −80°C. ELISA method was used (Desacyl−Ghrelin−ELISA Kit, SCETIMitsubishi Kagaku Iatron, (Tokyo, Japan) for measurement of the total plasma ghrelin levels. Inter− and intra−assay coefficients were 3.7% and 8.1%, respectively; the detection limit was 3.68 pg/mL. The ELISA method was also used for measurement of serum IGF−1 and IGFBP−3 levels (BIOSOURCE−ELISA Kit−Belgium).

**Statistical Analysis**

All values were analyzed in SPSS 16.0 (SPSS software for Windows, SPSS Inc, Chicago, IL) and showed a normal distribution according to the Kolmogorov−Smirnov test. Mann−Whitney U test was used to compare values between groups and Pearson’s correlation analysis−to investigate correlations between parameters. All values were expressed as “mean±standard deviation”. “p” values below 0.05 were accepted as significant.

## RESULTS

The mean chronological ages in both groups were similar, and the bone ages of children with CDGP were significantly lower than those of the control group. Although the mean weight and height values of children with CDGP were significantly lower than those of the control group, no significant difference was found in BMI values. The mean weight and height SDS values of children with CDGP were significantly lower than those of the control subjects; the mean BMI SDS values were similar in both groups (Table 1).

Table 2 shows the results for hormonal parameters in the two groups. Mean serum IGF−1 and IGFBP−3 levels of children with CDGP were lower than those of children in the control group (p=0.001 and p=0.003). In children with CDGP, IGF−1 and IGFBP−3 SDS levels calculated according to chronological age were significantly lower (p=0.0001 and p=0.0001), but they were similar when they were compared according to bone age (p=0.279 and p=0.114). No significant differences existed in both fasting and

postprandial ghrelin levels in children with CDGP and in children in the control group (p=0.427, p=0.112, respectively). The difference in ghrelin levels between fasting and postprandial states in children with CDGP was 394.44±369.10 pg/mL (the drop in ghrelin levels with feeding was 48%). In the control group, this difference was 346.55±338.67 pg/mL (the drop in ghrelin levels with feeding was 53%). The differences between fasting and postprandial levels of ghrelin were similar in both groups (p=0.885).

In a 3−day diet record, children with CDGP had significantly lower daily calorie intake compared to the control group (1250 kcal/day versus 1880 kcal/day).

**Correlations**

As seen in Table 3, fasting ghrelin correlated negatively with chronological age, bone age, height, weight, BMI and BMI SDS, and IGF−1. Postprandial ghrelin level negatively correlated only with weight, BMI and BMI SDS.

**Table 1:**
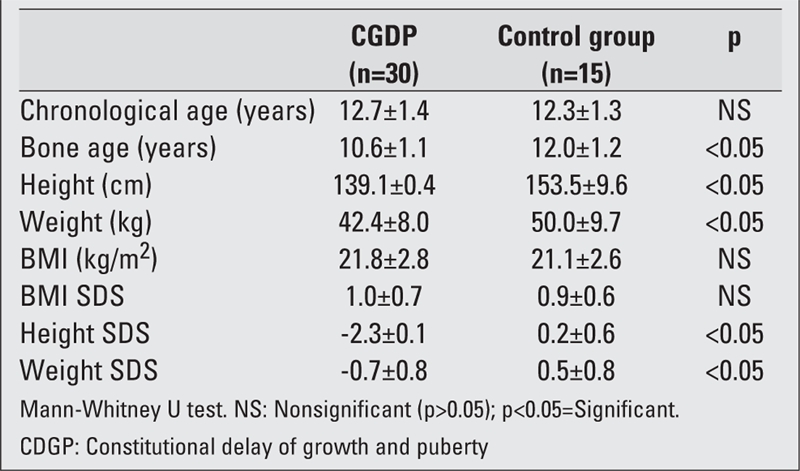
Anthropometric findings of the children with CDGP and of the control group

**Table 2:**
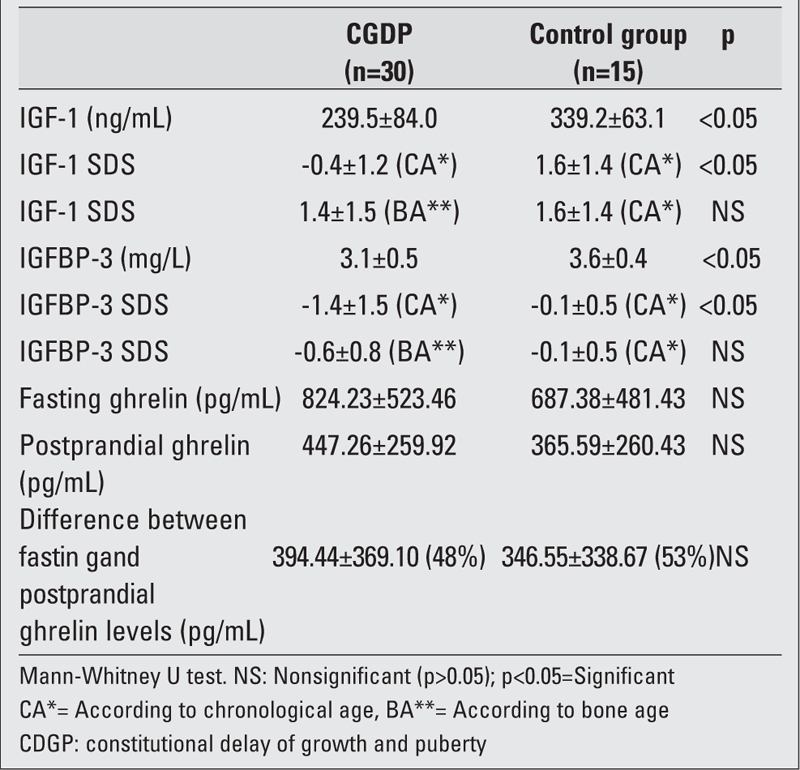
Mean ghrelin, IGF−1, IGFBP−3 levels and their SDS values of children with CGDP and in the control group

**Table 3:**
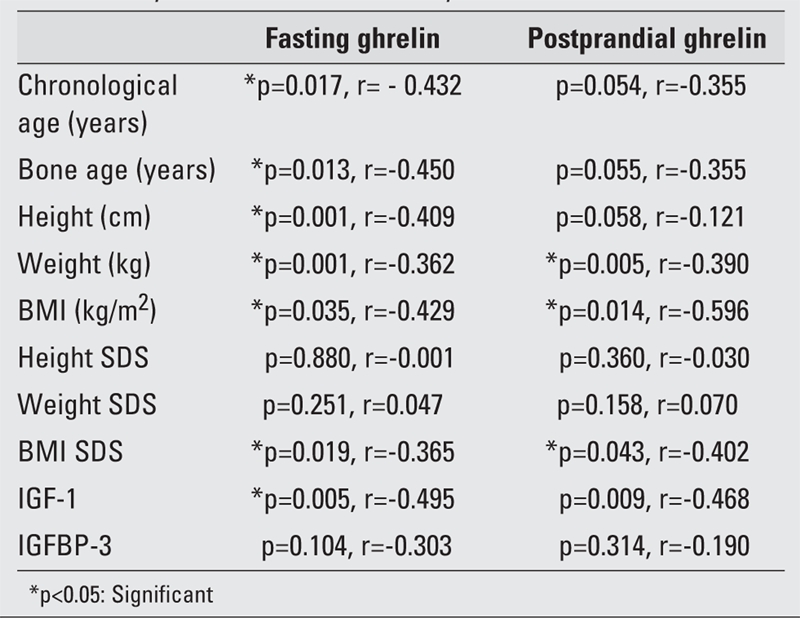
Fasting and postprandial ghrelin levels in children with CDGP, evaluation by Pearson correlation analysis

## DISCUSSION

The cause of growth delay in CDGP is not clear. The suggested role of partial and temporary functional hypopituitarism or partial and temporary GH deficiency has not been documented (12, 13, 14, 15). Since children with CDGP show a growth pattern similar to that of undernourished children, suboptimal nutrition from the early months of life was reported to be a cause of CDGP (16, 17). Horner et al (4) found that children with CDGP received an insufficient daily food intake, which provided them with fewer calories compared to children with normal growth pattern. Failure to thrive in the early months of infancy is reported to be present in most children with CDGP (4, 18, 19). In animals, suboptimal nutrition caused hormonal imbalance, which did not revert to normal following initiation of normal feeding (20). In rats, prolonged restriction of protein caused irreversible aberrations in GH−IGF−1 axis, which did not improve when the protein restriction ceased (20). In previous studies, it was reported that individuals who consumed a low−calorie diet had higher ghrelin levels compared to those who consumed a normal−calorie diet and advocated that higher ghrelin levels reflected a compensatory mechanism to increase food intake (21). Thus, we expected to find higher ghrelin levels in children with CDGP, who had lower daily calorie intake compared to the children in the control group. In a similar study, children with CDGP were found to have higher ghrelin levels than children with normal growth patterns, a finding, which was also interpreted to be a compensatory increase (22). In the present study, however, we found similar ghrelin levels in children with CDGP and in the control group, so we were not able to make any argument about the role of ghrelin in growth delay. We could have speculated that the fall in ghrelin levels with meals may be more prominent in children with CDGP, they got satiety more rapidly than normal children and consequently consumed less food, but our results do not support this explanation either. The differences between fasting and postprandial ghrelin levels were similar in children with CDGP and in the control subjects. We can only speculate that there may be insensitivity to ghrelin in children with CDGP.

Since CDGP is seen more commonly in males, our study group was composed of male children only. Weight and height SDS values of children with CDGP were lower than those of the control group. However, there were no significant differences in the BMI and BMI SDS values between the two groups. It is interesting that despite a low caloric intake, the BMI values of the 2 groups were similar. More in−depth and longitudinal studies may be needed to investigate the role of acute and chronic undernutrition in the etiology of CDGP. The similar BMI values in children with CDGP and in the control group might also explain the unchanged ghrelin levels found in this study.

Wudy et al (23) found lower BMI values in children with idiopathic short stature, and showed the importance of nutrition in growth delay. In that study, similar to our results, the authors reported no difference in ghrelin levels between children with idiopathic short stature and children with normal growth. Park et al (24) found negative correlations between ghrelin levels and anthropometric parameters such as weight, height, BMI, waist circumference and hip circumference in healthy children. In our study, we observed that both fasting and postprandial ghrelin levels correlated negatively to weight and BMI in children with CDGP. Whatmore et al (25) showed that ghrelin decreased with increased age. Our results were similar, but although a negative correlation between ghrelin and both chronological and bone ages was found in children with CDGP, such correlation was not detected in the control group. In our study, height, weight and BMI were the most important determinants of ghrelin levels in children with CDGP as well as in children in the control group.

Whatmore et al (25) defined IGF−1 and IGFBP−1 as the most important determining factors for ghrelin. In our study, ghrelin levels negatively correlated with IGF−1, but did not correlate with IGFBP−3. The negative correlation between ghrelin and IGF−1 may be related to the inhibitory effects of ghrelin on peripheral IGF−1 actions. In contrast to ghrelin, insulin levels are highest after meals and at lower levels just before meals. IGFBP−1 levels change in parallel to ghrelin levels (26). The dynamic interactions between ghrelin, insulin and IGFBP−1 show that ghrelin affects IGF−1 and insulin actions at tissue level. Thus, in our study, we found that as ghrelin levels rise, IGF−1 levels drop. In puberty, IGF−1 levels increase and IGFBP−1 levels decline, which is in parallel to increased growth (25, 26). These findings show us that ghrelin does not stimulate growth directly, but affects growth indirectly through its effect on the interactions within the IGF−1−GH axis (25, 26).

In our study, IGF−1, IGFBP−3 and their SDS values were lower in children with CDGP as compared to children in the control group. When we calculated IGF−1 and IGFBP−3 SDS values according to bone age, no difference was found in SDS values between the two groups. Evaluation of IGF−1, IGFBP−3 and their SDS values must be made according to bone age, rather than chronological age in children of pubertal ages.

Lower IGF−1 levels in children with CDGP were probably an outcome of suboptimal nutrition, rather than GH deficiency. Although ghrelin levels were similar in the study and the control groups, unidentified defects in ghrelin effect in children with CDGP might have caused growth delay indirectly by decreasing food intake. Our results are insufficient to clarify the role of ghrelin in growth delay in children and show that there are still many unknown points related to both ghrelin and CDGP.
